# The Book World of Medicine and Science

**Published:** 1900-10-27

**Authors:** 


### The Book World of Medicine and Science.

Manual of Surgery for Students and Practitioners. By William Rose, M.B., B.S. (Lond.), F.R.C.S., Professor of Clinical Surgery in King's College, London, and Senior Surgeon to King's College Hospital, and Albert Carless, M.S. (Lond.), F.R.C.S., Surgeon to King's College Hospital. Third edition. (London: Baillière, Tindall and Cox. 1900. Price 21s. net.)

The remarkable success which was at once achieved by this book is shown by the rapid call for new editions. Only twelve months have elapsed since the publication of the second edition, and already we find a third in our hands. Naturally the changes made are not great; but such as they are, they constitute considerable improvements. There is no doubt that the authors are right in thinking that to separate the chapters on the injuries of certain organs from those on their diseases was a mistake; and by bringing these together, so that the arrangement of the chapters is more according to regions than was the case before, the convenience of the book is much increased. Several new illustrations have been added, and altogether the matter has been brought up to date. We repeat the criticism which we made in regard to the last edition—namely, that some gynæcological operations are so purely surgical as to deserve more attention than they have received. No doubt a surgeon who is *au fait* at all that is told us about the surgery of the intestines and other abdominal organs would not find himself in any serious difficulty if called upon to deal with a ruptured tube or a twisted ovarian pedicle. Nevertheless there are points about such operations which we think might as well have been explained. However much the authors may have felt called upon to economise in pages, and for that purpose to leave gynæcology on one side, one cannot avoid a sense of lack of completeness in a chapter on abdominal surgery which leaves such subjects as we have alluded to untouched. But if we take the book with the limitations which the authors have themselves imposed on their undertaking by excluding certain specialties, there can be no doubt that this is an admirable manual, well arranged, easy to refer to, and thoroughly representative of the surgery of to-day. We would also say a word in praise of the printing and of the manner in which the matter is set out; the judicious use of darker type to head the important paragraphs, and to give emphasis to leading words, being a great help in referring back to any subject.

Tropical Diseases: A Manual of the Diseases of Warm Climates. By Patrick Manson, C.M.G., LL.D. (Aberd.). Revised and enlarged edition, 684 pages, with 114 illustrations. (London: Cassell and Company. 1900. Price 10s. 6d.)

We welcome the appearance of a second edition of Dr. Manson's well-known and widely-read book on Tropical Diseases. In this edition the changes made are chiefly in the form of additions to the original text, and among these the most valuable will be found in the chapters devoted to the subject of malaria. Taking them in the order they appear, we notice the following as chiefly worthy of attention:—Under the heading Zoological Affinities—the affinities of the plasmodium to which malarial fever in man has been traced—two pages are added, describing the parasites found in the blood of frogs, cattle, and birds, with drawings of these after Celli. Peculiar interest attaches to the infected birds from the fact that it was in studying the life history of a bird-parasite, the proteosoma, that definite knowledge regarding the rôle played by the mosquito in the cycle of its development and transmission was first obtained. In this edition Dr. Manson describes the mosquito-malarial theory as now definitely formulated, and gives a detailed summary of the important work done by Ross, of Liverpool, in connection with it; a summary which has its value enhanced by a series of drawings, illustrating the evolution of the crescent parasite, the appearances seen in the stomach of the mosquito after infection by proteosoma, and the anatomical details of the venulo-salivary gland, and its duct, through which the parasite of malaria is conveyed from the mosquito to man. Besides these drawings there are others showing the external appearances which distinguish Anopheles, the dangerous mosquito, so far as man is concerned, from the comparatively harmless Culex. In discussing pernicious fevers Dr. Manson draws attention to the rare accident of amblyopia in comatose cases, and gives a table in which the details of this eye affection are contrasted with those of a somewhat similar affection, said to follow the administration of large doses of quinine. The distinction is of considerable importance as regards treatment, as obviously quinine must at once be given in the one case, and at once withdrawn in the other. Dr. Manson gives Poncet as his authority for the ophthalmoscopic appearances seen in the case of pernicious fever.

The chapter on Filaria—the subject which Dr. Manson has made more peculiarly his own—is extended, and an addition made to our knowledge of the exact details of the phenomenon known as "filarial periodicity." This is given in the account of a post-mortem examination recently made in England on the body of a patient who at the time of death was suffering from lymph scrotum and varicose groin glands. The man committed suicide in the morning, at a time when the filarial embryos had almost disappeared from the blood in the surface of the body. The examination was made six hours after death. The average number of the embryos aggregated in the various internal organs was calculated from the contained blood and from sections of the tissues, and the result showed the lungs to contain by far the greater number. The facts are interesting, but the cause of the migration from the surface remains still unexplained. In the same chapter there are three new drawings representing filaria lying between the fibres of the thoracic muscles of the mosquito on the first, seventh, and twelfth days after the insect had fed on a patient suffering from filariasis. These are copies from microphotograms by Mr. Spitta, and are a decided improvement on the drawing illustrating the same condition in the first edition. In concluding this short notice of a book which we regard as a very valuable addition to the medical literature of the day, we have to congratulate Dr. Manson on the successful accomplishment of the task he has undertaken—that of bringing the subjects treated well up to date, and at the same time preserving the character of his book as being of convenient size and easily carried about by traveller.

